# The Employment of External Heat and Cold

**Published:** 1854-12

**Authors:** 


THE EMPLOYMENT OF EXTERNAL HEAT AND COLD.—In an interesting letter to the Editor of the *Bull. Gén. de Thér.*, (Sept. 15), M. Legroux states the opinions he has arrived at from the two measures above-named. He believes that the use of great degrees of heat is positively and considerably hurtful. Even moderate heat appears to do harm. "Moderate as it may be," says M. Legroux, "heat, whether applied externally, or in the form of hot drinks, augments the malaise and the anxiety. Few of the patients find relief. But when the heat is extreme, and produced by the agency of hot metal, bricks, bottles, hot bags, and when thick covertures, cushions, and eider down pillows are added, the anxiety becomes inexpressible, the dyspœna is extreme, the patient tosses about vainly, imprisoned as he is by his coverings, for the purpose of perceiving and breathing the cool air; the epigastric fire increases, and the cramps augment in the direct ratio of the heat. It is a veritable torture, a frightful torment. Great heat is fatal to patients with cholera. This fact is incontestable, and the public should be warned of it."

On the other hand, Legroux has found evident benefit from cold, iced drinks, from allowing the patient to roll freely in bed, so that the cool air may blow upon him.

The author speaks very highly of the effect of sinapisms. "To conclude," he says, "the fatal effect of an excessive calorification, the benefit of sinapisms, and the good effects of cold drinks, are the only therapeutical facts which it is possible to generalize in the confirmed algide cholera."—*British and Foreign Medico-Chirurgical Review.*

